# Supplementary material for: Hybrid Models and Biological Model Reduction with PyDSTool
Source: PLoS Comput Biol. 2012 Aug 9;8(8):e1002628. doi: 10.1371/journal.pcbi.1002628 (PMC3415397; doi:10.1371/journal.pcbi.1002628)
Supplement: Text S4 — Complete source code for the PyDSTool package (version 0.88.120504). Includes API documentation and help files linking to web pages. This file is identical to the current public release on Sourceforge.net. (ZIP) [file pcbi.1002628.s004.zip › PyDSTool/html/PyDSTool.Redirector-module.html]

xml version="1.0" encoding="ascii"?


PyDSTool.Redirector


| Home | Trees | Indices | Help | PyDSTool |
| --- | --- | --- | --- | --- |

Package PyDSTool ::
Module Redirector

[hide private]

[frames] | no frames]

# Module Redirector

source code

```
    Redirect stdout / stderr to temp file

 Originally by R. Kern, 2005
 Adapted by R. Clewley, 2006


Copyright (c) 2005 Robert Kern.

All rights reserved.

Redistribution and use in source and binary forms, with or without
modification, are permitted provided that the following conditions are met:

  a. Redistributions of source code must retain the above copyright notice,
     this list of conditions and the following disclaimer.
  b. Redistributions in binary form must reproduce the above copyright
     notice, this list of conditions and the following disclaimer in the
     documentation and/or other materials provided with the distribution.
  c. Neither the name of the Enthought nor the names of its contributors
     may be used to endorse or promote products derived from this software
     without specific prior written permission.


THIS SOFTWARE IS PROVIDED BY THE COPYRIGHT HOLDERS AND CONTRIBUTORS "AS IS"
AND ANY EXPRESS OR IMPLIED WARRANTIES, INCLUDING, BUT NOT LIMITED TO, THE
IMPLIED WARRANTIES OF MERCHANTABILITY AND FITNESS FOR A PARTICULAR PURPOSE
ARE DISCLAIMED. IN NO EVENT SHALL THE REGENTS OR CONTRIBUTORS BE LIABLE FOR
ANY DIRECT, INDIRECT, INCIDENTAL, SPECIAL, EXEMPLARY, OR CONSEQUENTIAL
DAMAGES (INCLUDING, BUT NOT LIMITED TO, PROCUREMENT OF SUBSTITUTE GOODS OR
SERVICES; LOSS OF USE, DATA, OR PROFITS; OR BUSINESS INTERRUPTION) HOWEVER
CAUSED AND ON ANY THEORY OF LIABILITY, WHETHER IN CONTRACT, STRICT
LIABILITY, OR TORT (INCLUDING NEGLIGENCE OR OTHERWISE) ARISING IN ANY WAY
OUT OF THE USE OF THIS SOFTWARE, EVEN IF ADVISED OF THE POSSIBILITY OF SUCH
DAMAGE.
```


|  |  |
| --- | --- |
| Classes | [hide private] |

Redirector


|  |  |
| --- | --- |
| Variables | [hide private] |

STDOUT = `1`

STDERR = `2`

| Home | Trees | Indices | Help | PyDSTool |
| --- | --- | --- | --- | --- |

|  |  |
| --- | --- |
| Generated by Epydoc 3.0.1 on Fri May 4 15:24:03 2012 | http://epydoc.sourceforge.net |
